# Psychosocial Factors Influencing Parents’ Acceptance of COVID-19 Vaccination for Their Children: An Italian Cross-Sectional Study

**DOI:** 10.3390/vaccines12030317

**Published:** 2024-03-17

**Authors:** Miriam Capasso, Marcella Bianchi, Daniela Caso

**Affiliations:** Department of Humanities, University of Naples “Federico II”, 80133 Naples, Italy; marcella.bianchi@unina.it (M.B.); caso@unina.it (D.C.)

**Keywords:** theory of planned behavior, decision-making, vaccination intention, trust, COVID-19

## Abstract

Vaccine hesitancy poses a significant threat to the health of individuals across all age groups, which has been exacerbated by the COVID-19 pandemic. In this cross-sectional study, an extension of the Theory of Planned Behavior (TPB) was applied to investigate psychosocial variables predicting intention to vaccinate children under 12 against COVID-19 in a sample of 420 Italian parents (Mean age = 40.4, *SD* = 5.9; Women = 78.1%). Hierarchical regression analysis revealed that, among the TPB variables, cognitive attitude, descriptive norms, and perceived behavioral control significantly predicted parents’ vaccination intention. Furthermore, including trust in the institutions’ ability to manage the vaccination campaign in the model significantly increased the explained variance in intention. These findings suggest that campaigns promoting childhood COVID-19 vaccination should not only emphasize the safety and effectiveness of vaccines for children but also focus on reducing barriers to vaccination. Additionally, attention should be given to enhancing the perception that this behavior is widespread among other parents, thus leveraging the power of social influence. Finally, and not less important, significant efforts should be directed toward building and reinforcing trust in the system of actors promoting and managing the COVID-19 vaccination campaign.

## 1. Introduction

Vaccines stand among the most effective and secure interventions for the primary prevention of numerous infectious diseases. As is widely acknowledged, the introduction of vaccines has drastically reduced the likelihood of falling prey to dangerous and often debilitating illnesses and, in some instances, led to completely eradicating certain diseases (such as smallpox). Consequently, vaccination has been defined as a public good, a right every citizen should benefit from [1]. The global COVID-19 pandemic has further emphasized the critical role of vaccination in safeguarding public health. Despite the pandemic being declared over in May 2023 [2], vaccines keep on functioning as effective tools in controlling COVID-19 infection and mortality rates [3].

In Italy, the national COVID-19 vaccination campaign officially started on 27 December 2020, marking the so-called “vaccine day”. Following the prioritization outlined in the National Strategic Plan by the Ministry of Health, the initial recipients were healthcare workers, older adults, individuals with high vulnerability and their caregivers, and people under 60 with comorbidities. Once the priority categories were covered, the vaccination rollout was extended to the rest of the population, including children under 12 [4]. At the time of writing (February 2024), the Italian Ministry of Health [5] recommends a booster dose of the vaccine adapted to the Omicron XBB.1.5 variant for all people aged six months and older with conditions of health vulnerability exposing them to a higher risk of a more severe form of COVID-19. Nevertheless, vaccination is also available upon request to those who do not fall into the risk categories, including children.

Looking at trends in vaccination adherence, the participation rate in the Italian anti-COVID-19 vaccination campaign has steadily increased, reaching around 90% of the population over 12 who completed the primary vaccination cycle [6]. However, to date, the percentages of vaccinated children under 12 are worryingly low: only 35.37% of the 5–11 age group have completed the primary vaccination cycle, and merely 0.46% of the population eligible for an additional dose has received it. Additionally, for the 0–4 age group, only 0.03% of the population has received at least one dose of the vaccine. Indeed, despite the effectiveness of vaccines, vaccine hesitancy—i.e., «a delay in acceptance or refusal of vaccination despite the availability of vaccination services» [7] (p. 4163)—has emerged as a significant barrier to the success of the vaccination campaign, particularly among parents [8]. 

Vaccine hesitancy is a rather complex and multifaceted phenomenon. Indeed, the decision to vaccinate (or get vaccinated) can vary considerably based on the specific vaccine or whether it involves deciding for one’s health or others (for example, one’s children’s health). For instance, an individual may exhibit hesitation towards a newly developed vaccine due to concerns about its safety or efficacy, even if they generally support established vaccination practices. Similarly, the decision-making process may differ when considering the vaccination of children. Indeed, given the multidimensional nature of well-being [9,10], parents navigate through several considerations when making decisions about their children’s health. The decision to vaccinate becomes not just a matter of physical health but extends to social, emotional, and psychological dimensions. Therefore, vaccine hesitancy can be better understood when viewed as a “spectrum” of beliefs rather than a simple opposition between choosing to vaccinate (or get vaccinated) or not [11].

Research on factors predicting acceptance or refusal of COVID-19 vaccines began even before the approval of the first vaccine. However, few studies have delved into the variables shaping parents’ intention to vaccinate their children. Exploring this aspect is crucial, as the decision-making process leading to the choice of self-vaccination does not necessarily align with decisions regarding one’s children. To address this gap in the literature, this study aimed to apply an extended version of the Theory of Planned Behavior (TPB [12]) to comprehend parents’ intentions to vaccinate their children against COVID-19. It is worth noting that, despite being conducted in the midst of the pandemic, the study findings remain relevant in the post-pandemic era. This is because, even though the emergency has ended, the virus continues to cause a significant number of deaths worldwide, including among children [13].

### Theoretical Framework

The TPB [12] is grounded on the assumption that the most proximal predictor of behavior is represented by behavioral intention. This core component, in turn, is determined by three conceptually independent factors: attitude, subjective norms, and perceived behavioral control (PBC). Attitude refers to the favorable or unfavorable evaluation of a particular behavior, including both a cognitive component (e.g., “Vaccinating children against COVID-19 is safe”) and an affective component (e.g., “Vaccinating children against COVID-19 is pleasant”). On the other hand, subjective norms reflect the perception of social pressure from significant others (or social groups to which the individual feels a sense of belonging) to engage or not engage in a particular action and the personal motivation to conform to these expectations. Subjective norms can also be distinguished into two types [14]: injunctive norms, indicating the perception of pressure from significant others to adopt a certain behavior, and descriptive norms, signifying the perception that significant others actually adopt that behavior. Lastly, PBC can be defined as the extent to which an individual perceives having the abilities and/or control to perform the behavior and can predict it directly and indirectly (via intention). A large body of research has confirmed the high predictability of the TPB in various health domains [15,16,17,18,19].

Numerous studies have also employed this theoretical framework to predict the intention and actual adherence to various types of vaccinations, including that against COVID-19 [20,21,22,23,24]. In this regard, a meta-analysis conducted by Limbu and colleagues [25] revealed that attitude exhibited the most robust correlation with COVID-19 vaccination intention (*r* = 0.487), followed by subjective norms (*r* = 0.409) and perceived behavioral control (*r* = 0.286). Thus, the more favorably vaccination is evaluated (in particular, in terms of safety, efficacy, and utility), the more it is perceived that getting vaccinated (or vaccinating) is a socially approved behavior by significant others or other social groups important to the individual, the higher the vaccination intention becomes. Moreover, a greater sense of control over the vaccination decision-making process contributes positively to the intention to get vaccinated.

Despite the proven effectiveness of the TPB model in predicting and explaining vaccination intentions and related behaviors, its application to the understanding of parents’ decisions to vaccinate their children against COVID-19 is notably limited. In the aforementioned meta-analysis [25], parents accounted for only 4.7% of the examined population, and none of the included studies were conducted in Italy (No additional Italian studies were found in our search even after the publication date of the meta-analysis by Limbu and colleagues). Therefore, this study aimed to fill this research gap by focusing on Italian parents’ vaccination intentions. In doing so, we expanded our examination beyond traditional TPB variables to include the influence of trust in institutions—a key factor in the general domain of preventive behaviors [26,27,28], which has also been recognized as one of the most critical predictors of vaccination acceptability [29,30]. In particular, trust in institutions is based on the perception that institutions possess the necessary capabilities and resources to carry out their assigned tasks and the belief that they work to maximize people’s health [31]. To an even higher level of specificity, high levels of trust in institutions’ ability to manage pandemic events have been associated with the adoption of protective health behaviors [32], highlighting the need to build and maintain trust relationships with institutions before the outbreak of potential pandemics [33].

Recently, this relationship has been confirmed in studies focusing on COVID-19 vaccination. For instance, in a study about the predictors of negative attitudes towards the COVID-19 vaccine involving English adults, Paul and colleagues [34] showed that low levels of trust in institutions’ ability to manage the pandemic were associated with greater distrust in the safety characteristics of the vaccine and more significant concerns about vaccination side effects. Relatedly, in a study across 32 countries—including Italy—the belief that the government was handling the pandemic well was associated with greater vaccine acceptance in most examined countries [35]. Moreover, several studies have integrated such a variable into the TPB model. For example, in a study integrating the Theory of Planned Behavior with the Health Belief Model [36], Patwary et al. [37] evidenced that trust in institutions significantly predicted the acceptability of the COVID-19 vaccination beyond the effect of the other variables in the integrated model. Similarly, Knobel et al. [38], using a two-step cluster analysis, found that the cluster of individuals most skeptical towards the COVID-19 vaccination was characterized by lower intentions, less favorable attitudes, lower subjective norm, and less trust in the government’s commitment to safeguarding the well-being of its citizens in managing the pandemic.

Starting from this theoretical framework, the present research aimed to explore the role of TPB variables plus trust in institutions in predicting parents’ intention to vaccinate their children against COVID-19. Specifically, we hypothesized that intention would be positively predicted by cognitive attitude (H1), affective attitude (H2), injunctive norms (H3), descriptive norms (H4), and perceived behavioral control (H5). Moreover, we expected that the inclusion of trust in institutions within the TPB model would have significantly increased the explained variance in parents’ vaccination intention (H6).

## 2. Materials and Methods

### 2.1. Procedure and Participants

The research was conducted online using the “Google Forms” platform, and the questionnaire link was shared through informal channels (e.g., social network groups). To be eligible to participate in this study, participants had to meet the following criteria: (1) be of legal age (age > 18) and (2) have at least one child under 12 not yet vaccinated against COVID-19.

Before proceeding with the recruitment of participants, we carried out, using G*Power [39], an a priori power analysis to estimate the required sample size for detecting a medium effect size (f^2^ = 0.15) for a hierarchical regression analysis with 7 psychological predictors and 15 socio-demographic predictors (as explained below), an alpha = 0.05, and power = 0.80. The estimated sample size was *N* = 163. Among the invited participants, 420 parents met the inclusion criteria and completed the questionnaire after being informed of the anonymity of the data collection and giving informed consent. Therefore, the sample size appears more than adequate for testing the statistical hypotheses.

Participants were predominantly mothers (78.1%), married or in a romantic relationship (79%), and had one or two children (90%). Their youngest child had an average age of 6.5 years (*SD* = 3). Moreover, the majority held a high school diploma or a degree (91.2%), reported being in a middle socio-economic status (70.5%), and identified as Catholic (80.5%). Regarding political orientation, 37.9% described themselves as apolitical, 28.6% identified as left-wing, 14.5% as right-wing, 10.2% as center, and the remaining 8.8% declared different orientations. Concerning vaccination behavior, the majority of parents (89%) received the COVID-19 vaccination (with two doses) and other recommended vaccinations, such as the HPV vaccination (73.6%). However, only 24.8% received the flu vaccination. Regarding experience with COVID-19, 18.6% of the parents declared having contracted the virus, and 17.4% stated that at least one of their children tested positive for COVID-19. Finally, 6% of parents reported that their under-12 child(ren) suffered from specific health problems (e.g., allergies, cardiovascular problems, or neurodevelopmental disorders).

### 2.2. Measures

In the initial section of the questionnaire, participants completed the informed consent form. Subsequently, they were informed that upcoming sections would have included questions about their views on COVID-19 vaccination in children under 12. Consequently, they were instructed to answer these questions considering their son or daughter under 12. If they had more than one child in this age group, they were requested to think about their children in this age range in general. Following this instruction, the subsequent measures were administered to all participants in the same order. All TPB items were developed by referring to the guidelines formulated by Ajzen [40].

Parents’ intention to vaccinate their children against COVID-19 was measured with three items on a 5-point Likert scale from completely disagree (1) to completely agree (5) (e.g., “I intend to vaccinate my child/children against COVID-19”). Cronbach’s α = 0.98.

Parents’ attitude toward vaccinating their children against COVID-19 was assessed through five items on a semantic differential scale ranging from 1 (negative pole) to 5 (positive pole). The first three items focused on the cognitive component (e.g., “Vaccinating my child[ren] against COVID-19 would be harmful/beneficial”), whereas the other two items focused on the affective dimension (e.g., “Vaccinating my child[ren] against COVID-19 would be unpleasant/pleasant”). Cronbach’s α = 0.95 for cognitive attitude and α = 0.89 for affective attitude.

Parents’ subjective norms were evaluated with four items on a 5-point Likert scale from completely disagree (1) to completely agree (5). Two items focused on injunctive norms (e.g., “Most people important to me think I should vaccinate my child[ren] against COVID-19”), and the other two on descriptive norms (e.g., “Most of the people important to me have vaccinated/will vaccinate their child[ren] under 12 against COVID-19”). Cronbach’s α = 0.94 for injunctive norms and α = 0.80 for descriptive norms.

Parents’s perceived behavioral control was assessed through three items on a 5-point Likert scale from completely disagree (1) to completely agree (5) (e.g., “Vaccinating my child[ren] against COVID-19 is entirely up to me”). Cronbach’s α = 0.72.

Trust in institutions was measured using six items adapted from Trent et al. [41]. Specifically, we used three items for evaluating general institutional trust (i.e., “How much do you trust international political organizations?”, “How much do you trust your national government?”, and “How much do you trust your regional government?”), and three items for measuring trust in institutions’ ability to manage the vaccination campaign (i.e., “How much do you trust the decisions of international political organizations regarding COVID-19 vaccination in children?”, “How much do you trust the decisions of your national government regarding COVID-19 vaccination in children?”, and “How much do you trust the decisions of your regional government regarding COVID-19 vaccination in children?”). All items were rated on a 5-point scale from not at all (1) to very much (5). Cronbach’s α = 0.90 for general institutional trust and α = 0.96 for trust related to vaccination campaign management.

In the last section of the questionnaire, we asked parents to indicate the following socio-demographic characteristics: age, gender, youngest children’s age, number of children, marital status, socio-economic condition, education, religious orientation, political orientation, past vaccination behavior (adherence to COVID-19 vaccination, flu vaccination and other recommended vaccinations), having personally or own children tested positive for COVID-19, and whether children suffered from specific health problems.

### 2.3. Data Analysis

Statistical analyses were conducted using SPSS 29. Firstly, descriptive analyses were carried out on all study variables. Moreover, zero-order correlations were estimated to evaluate the associations between psychological variables. 

Subsequently, a three-step hierarchical multiple regression analysis was performed to test the significance of predictors in the extended TPB model. Based on Barbaranelli’s recommendations [42], we first examined the following assumptions of regression analysis: (1) linearity of the relationship between the independent variables and dependent variable; (2) absence of multicollinearity among the independent variables; and (3) normality distribution of error terms (residuals). The first assumption was validated by visually inspecting scatterplots of the dependent variable plotted against each independent variable. Secondly, analysis of the collinearity statistics showed that each predictor had tolerance values greater than 0.20 and VIF coefficients less than 5.0, indicating the absence of several multicollinearity issues [43]. Finally, the assumption of normality in the distribution of residuals was confirmed by checking that the graphs depicting the distribution of residuals (histogram and P–P plot) were consistent with normality. 

Following the examination of these assumptions, we proceeded by including the variables in the regression model in the following order: socio-demographic control variables, which were coded into dummy variables (Model 1); TPB variables, i.e., cognitive attitude, affective attitude, injunctive norms, descriptive norms, and PBC (Model 2); general institutional trust and trust in institutions’ ability to manage the vaccination campaign (Model 3). A significant F change (*p* < 0.05) implies that the added variables significantly improve the model prediction. 

All the answers to the questionnaire were mandatory, so there were no missing values.

## 3. Results

### 3.1. Descriptive Analysis

Table 1 shows descriptive analysis and zero-order correlations between the psychological variables. Participants showed moderate levels across all TPB constructs, with the highest mean observed for cognitive attitude. On the other hand, general institutional trust and specific trust in their ability to manage the vaccination campaign levels were below the mean. All variables were positively and significantly correlated.

### 3.2. Regression Analysis

The results of the regression analysis (Table 2) showed that the socio-demographic variables alone (Model 1) accounted for 27% of the variance in intention. Specifically, having received COVID-19, flu, and other recommended vaccinations, as well as having higher education, positively influenced intention to vaccinate children. Conversely, identifying as Catholic had a negative impact. Upon introducing TPB variables (Model 2), there was a substantial increase in the explained variance, reaching R^2^ = 73%. Within the TPB constructs, cognitive attitude, descriptive norms, and PBC emerged as significant and positive predictors of intention. Finally, the addition of trust variables (Model 3) led to a further and significant increase in explained variance, with R^2^ = 75%. Consequently, the integrated model could be considered the best-fitting one. Notably, in this final model, only trust in the ability of institutions to manage the vaccination campaign—but not general institutional trust—significantly predicted parents’ vaccination intention.

For exploratory purposes, we also tried to introduce general and specific institutional trust in two subsequent steps. Interestingly, in the model including socio-demographic covariates, TPB variables, and general institutional trust, the latter significantly predicted vaccination intention (β = 0.09, *p* = 0.006). However, upon introducing specific institutional trust in the final model, it emerged as the only significant predictor of intention (β = 0.24, *p* = 0.000), while general trust no longer remained significant (β = −0.05, *p* = 0.286).

## 4. Discussion

The aim of this study was to test the efficacy of an extended version of the Theory of Planned Behavior [40] in predicting parents’ intention to vaccinate their children against COVID-19. While numerous studies have supported the validity of this model in predicting COVID-19 vaccination intention in the general population, none have specifically investigated parents’ intentions in the Italian context.

Overall, our results revealed that parents had moderate levels in all TPB variables and below-average levels of trust, both in institutions in general and in their ability to manage the vaccination campaign. In comparison to findings of studies on adults (e.g., [44]), vaccination intentions appear to be at lower levels. This observation could be explained by the fact that parents may exhibit higher levels of hesitancy towards COVID-19 vaccination for their children compared to their own vaccination. In this regard, a recent meta-analysis by Bianchi et al. [45], analyzing parental COVID-19 vaccine hesitancy in the Italian context, found that 55% of parents were hesitant to vaccinate their children. The main reasons included a lack of information about vaccination (especially from healthcare professionals), fear of adverse events, and the idea that the vaccine is not safe and/or effective for children.

As for the main analyses, the results showed that in the best-fitting model (Model 3), among socio-demographic variables, only the age of the youngest child and the parents’ COVID-19 vaccination status positively predicted the parents’ intention to vaccinate their children. This aligns with the aforementioned meta-analysis [45], showing that the older age of the child was associated with higher vaccine compliance, possibly due to the idea that older children are less likely to experience vaccine side effects. Concerning the impact of parents’ vaccination status, Bianchi and colleagues proposed that personally receiving the COVID-19 vaccine could serve as a potentially positive predictor. This is because not experiencing side effects firsthand might make individuals more inclined to vaccinate their children.

In relation to the antecedents of intention, traditional TPB variables accounted for a substantial portion of the variance in the intention to vaccinate children (73%). Specifically, cognitive attitude, descriptive norms, and PBC were significant predictors, confirming H1, H4, and H5. However, the impact of affective attitude and injunctive norms was not confirmed; thus, H2 and H3 were not supported. Notably, attitude emerged as the most influential among these factors, consistent with findings from studies conducted on general COVID-19 acceptance [22,23,46,47].

Concerning attitude, the significance of the cognitive component alone in predicting intention aligns with research suggesting that vaccine choices are more likely linked to assessments of safety and efficacy rather than the emotional appeal of the behavior [48]. However, it is essential to underline that the affective component of attitude is not inconsequential in vaccination choices. While the emotions associated with performing this behavior may not be influential in deciding to implement it, expecting to feel specific emotions after vaccination—e.g., pride or relief for having protected themselves and others—may instead play a significant role [49,50,51]. Thus, future studies should investigate whether anticipated emotions can also influence parents’ choices to vaccinate their children against COVID-19.

Regarding subjective norms, it is interesting to note that the lack of a significant relationship between injunctive norms and intention contrasts with results from prior studies on the general population, which showed that the decision to vaccinate against COVID-19 can be strongly influenced by pressure from significant others [21,52,53]. However, many of these studies did not differentiate between injunctive and descriptive components of norms, which may explain why, in our findings, only descriptive norms influenced parental intentions. In fact, vaccination compliance is higher when one believes this behavior to be widespread among one’s peers [54], potentially regardless of the perceived pressure to do so. Applying this line of reasoning to our participants, it is likely that the perception of other parents choosing to vaccinate their children contributes to the idea that this is a “normal” and widespread behavior, influencing their own intentions.

Moreover, the positive relationship identified between perceived behavioral control and intention suggests that the perception of having control over vaccinating one’s children is crucial in shaping the decision to vaccinate them. This result becomes particularly noteworthy considering the mixed findings reported in the literature regarding the impact of PBC on personal vaccination intentions against COVID-19, as some studies found a significant relationship (e.g., [22]), whereas others have not (e.g., [44]). In the present study, the significance of PBC in predicting parental intentions may be attributed to the unique context of decision-making for children’s vaccination. Parents might perceive a greater sense of responsibility and control when it comes to deciding on their children’s health—especially in relation to COVID-19 protective behaviors [55]—thus amplifying the importance of PBC in shaping their vaccination intentions.

As hypothesized (H6), the inclusion of trust in institutions within the TPB model significantly increased the explained variance in parents’ vaccination intention. Interestingly, only trust in the institutions’ ability to manage the vaccination campaign for children, rather than general institutional trust, influenced parents’ intention. This finding aligns with studies on past pandemics (e.g., influenza H1N1 outbreak [32,56]) and the COVID-19 pandemic [33], highlighting that specific confidence in the institutions’ ability to manage the emergency positively impacts vaccination acceptability. By evaluating an even more specific type of trust, our study expands on the existing literature by showing that the more parents believe in the effective organization of the campaign by political institutions at regional, national, and international levels, the higher their intention to vaccinate their children becomes. This implies that efforts to enhance general trust in institutions in promoting this behavior may prove futile if they do not concurrently emphasize trust in the institutions’ competence to manage children’s vaccination campaigns.

Several limitations of this study should be acknowledged. Firstly, the use of a convenience sample limits the applicability of the findings to the broader population of Italian parents. The overrepresentation of mothers in the sample introduces a gender imbalance, potentially impacting the generalizability of the results to fathers. Additionally, the high educational level of participants (91.2% with a high school diploma or degree) may skew the results, as this demographic group might be more inclined towards vaccination. Furthermore, the correlational design employed in this study excludes the possibility of establishing a causal relationship between variables. For this reason, further experimental studies are required to confirm the directionality of the investigated relationships within the extended TPB framework. Another limitation is the focus on intention, thus the absence of a behavioral measure. Adopting a longitudinal design could clarify whether, over time, vaccination intention translates into actual behavior. Finally, the focus on trust was specific to government institutions, and future research could expand on this by comparing various types of trust, such as trust in scientists and healthcare professionals, to deepen understanding of the decision-making process leading (or not leading) parents to vaccinate their children against COVID-19.

## 5. Conclusions

The results of this study support the effectiveness of the TPB model, along with trust in institutions, in predicting Italian parents’ intention to vaccinate their children against COVID-19. This has implications both in theory and practice.

From a theoretical standpoint, our findings align with previous studies conducted on the general population [44] and also support the applicability of this model to understand parents’ vaccination intention. Additionally, including and confirming the impact of trust in the government’s ability to manage the vaccination campaign for children represents a point of novelty of this work, since most of the literature on this topic focused on general institutional trust or trust in their ability to manage the pandemic.

Practically, the findings provide valuable insights regarding the psychological factors to be addressed in upcoming COVID-19 vaccination promotion campaigns for children. First, the key role of cognitive attitude that emerged from the analyses suggests the importance of communicating the safety and effectiveness of COVID-19 vaccines for children. Tailored campaigns addressing specific parental concerns about childhood vaccination, especially for younger children, are essential in this context. Moreover, the observed significance of descriptive norms in predicting parents’ intention to vaccinate their children underscores the role of social norms in shaping individual behavior. In the context of childhood vaccination, it implies that interventions could strategically emphasize and portray it as a widely accepted and practiced behavior among other parents; for example, by incorporating real stories and testimonials [57] from parents who have chosen to vaccinate their children.

Regarding perceived behavioral control, interventions aimed at boosting parents’ confidence and addressing barriers in the vaccination process can significantly increase vaccination intentions. Providing clear information, simplifying procedures, and offering support for logistical challenges can contribute to enhancing their PBC. This becomes even more crucial with evolving vaccination recommendations, such as booster doses or modified vaccines, where a sense of control may play a key role in parents’ willingness to adapt to new guidelines and information [58].

Finally, our results emphasize the importance of building trust in the effectiveness and efficiency of the vaccination campaign. Simply enhancing the perception of institutions as credible and reliable is insufficient without concurrently increasing the belief that these institutions manage the vaccination campaign effectively [33].

In conclusion, as society transitions into the post-pandemic era, the insights derived from this study provide guidance for continuing to promote childhood COVID-19 vaccination and carry significant implications for pediatric public health. Future initiatives should draw on the lessons learned during the COVID-19 pandemic to establish effective strategies promoting the acceptance of childhood vaccinations. These strategies may include health promotion campaigns designed to reinforce trust in vaccine efficacy and, simultaneously, in the institutions managing vaccination campaigns. Such trust is crucial not only for building a prepared society in the event of future health challenges but also for combatting vaccine hesitancy in a broader and more multifaceted sense.

## Figures and Tables

**Table 1:** Descriptive analysis and correlations among the psychological variables.

	M (SD)	1.	2.	3.	4.	5.	6.	7.	8.
1. Intention	3.09 (1.46)	1							
2. Cognitive attitude	3.30 (1.34)	0.82 **	1						
3. Affective attitude	2.86 (1.32)	0.73 **	0.83 **	1					
4. Injunctive norms	2.96 (1.20)	0.68 **	0.67 **	0.63 **	1				
5. Descriptive norms	2.89 (1.17)	0.71 **	0.68 **	0.65 **	0.83 **	1			
6. PBC	3.24 (1.09)	0.51 **	0.49 **	0.51 **	0.43 **	0.47 **	1		
7. General institutional trust	2.33 (0.91)	0.56 **	0.57 **	0.49 **	0.44 **	0.43 **	0.37 **	1	
8. Trust in institutions—vaccination campaign	2.32 (1.07)	0.73 **	0.72 **	0.64 **	0.57 **	0.58 **	0.46 **	0.81 **	1

Note. ** *p* < 0.01.

**Table 2:** Hierarchical regression results.

Independent Variables	Model 1 β	Model 2 β	Model 3 β
Step 1: Socio-demographic variables			
Age	0.04	−0.02	−0.04
Youngest children’s age	0.07	0.05	0.06 *
Gender	−0.01	0.04	0.04
Number of children	−0.05	0.00	0.00
COVID-19 vaccination	0.37 ***	0.10 **	0.08 **
Flu vaccination	0.22 ***	0.03	0.03
Other recommended vaccinations	0.11 *	0.01	0.01
Socio-economic status	−0.02	0.01	0.01
Marital status	−0.04	−0.01	−0.02
Education	0.11 *	0.04	0.03
Political orientation	−0.08	−0.02	−0.01
Religious orientation	−0.09 *	−0.06 *	−0.05
Having tested positive for COVID-19	0.01	0.02	0.01
Children tested positive for COVID-19	0.01	0.05	0.05
Children’s health problems	−0.04	0.00	−0.01
Step 2: TPB variables			
Cognitive attitude	-	0.48 ***	0.39 ***
Affective attitude	-	0.06	0.04
Injunctive norms	-	0.07	0.07
Descriptive norms	-	0.20 ***	0.17 ***
PBC	-	0.11 **	0.08 **
Step 3: Additional variables			
General institutional trust	-	-	−0.05
Trust in institutions—vaccination campaign	-	-	0.24 ***
F-statistics	11.05(_15,404_) ***	57.88(_20,399_) ***	57.73(_22,397_) ***
Adjusted R^2^	0.27	0.73	0.75
Δ*R*^2^	-	0.45	0.02
Δ*F*	-	140.95 ***	15.16 ***

Note. *** *p* < 0.001; ** *p* < 0.01; * *p* < 0.05. Categorial demographic variables were dummy coded as follows. Gender: 1 = women, 0 = other; COVID-19 vaccination: yes = 1, no = 0; Flu vaccination: yes = 1, no = 0; Other recommended vaccinations: yes = 1, no = 0; Socio-economic status: middle and high = 1, other = 0; Marital status: married or in a relationship = 1, other = 0; Education: degree or higher qualification = 1, other = 0; Political orientation: apolitical = 1, other = 0; Religious orientation: Catholics = 1, other = 0; Having tested positive for COVID-19: yes = 1, no = 0; Children tested positive for COVID-19: yes = 1, no = 0; Children’s health problems: yes = 1, no = 0.

## Data Availability

The dataset that supports the findings of this study is available from the corresponding author upon reasonable request.
